# Glibenclamide in aneurysmatic subarachnoid hemorrhage (GASH): study protocol for a randomized controlled trial

**DOI:** 10.1186/s13063-019-3517-y

**Published:** 2019-07-09

**Authors:** Bruno Braga Sisnando da Costa, Isabela Costola Windlin, Edwin Koterba, Vitor Nagai Yamaki, Nícollas Nunes Rabelo, Davi Jorge Fontoura Solla, Manoel Jacobsen Teixeira, Eberval Gadelha Figueiredo

**Affiliations:** Neurosurgery Department, University of São Paulo, São Paulo, SP Brazil

**Keywords:** Subarachnoid hemorrhage, Brain aneurysms, Glibenclamide

## Abstract

**Background:**

Recent findings on the benefits of glibenclamide as a neuroprotective drug have started a new era for prospective studies on sulfonylureas. The effect of glibenclamide blocking the Sur1-Trpm4 channel was examined in models of subarachnoid hemorrhage and stroke, with findings of significantly reduced tight-junction abnormalities, resulting in less edema formation and considerably reduced transsynaptic apoptosis of hippocampal neurons and significantly ameliorated impairments in spatial learning. Based on these data, we plan a clinical trial to establish evidence of glibenclamide as an adjunct treatment in aneurysmal subarachnoid hemorrhage.

**Methods:**

An estimated 80 patients meeting the inclusion criteria of radiological confirmatory evidence of an aneurysmal subarachnoid hemorrhage, age 18–70 years, and presentation of less than 96 h from the ictus will be allocated randomly into two groups, one receiving 5 mg daily oral intake of glibenclamide for 21 days and another control group receiving a placebo. The study’s primary outcome is the modified Rankin scale (mRS) after 6 months, as favorable (mRS 0–2) or unfavorable (mRS 3–6). The secondary outcomes will be late cognitive status, assessed after 6 months by psychological tests (the Short Form Health Survey Questionnaire and the Montreal Cognitive Assessment), as well as death at 6 months, delayed cerebral ischemia and occurrence of serious adverse events due to study medication.

**Discussion:**

There is a growing interest in the scientific community regarding glibenclamide in brain edema and traumatic brain injury, but with very little of this interest targeting spontaneous brain hemorrhage, especially aneurism rupture. Positive outcomes are expected for the treatment patients, especially in language and memory preservation, as has been shown in experimental models.

**Trial registration:**

ClinicalTrials.gov, NCT03569540. Retrospectively registered on 26 June 2018.

**Electronic supplementary material:**

The online version of this article (10.1186/s13063-019-3517-y) contains supplementary material, which is available to authorized users.

## Background

Subarachnoid hemorrhage from a ruptured intracranial aneurysm is a common type of stroke and is of substantial clinical and socioeconomic relevance, affecting about 600,000 patients worldwide every year. Subarachnoid hemorrhage accounts for a great deal of death and disability, particularly because it affects a younger age group than other stroke subtypes. Combined morbidity and mortality reach up to 50%, with 30% of survivors remaining dependent. Additionally, 20–30% of patients in hospital admission, initially with a good clinical grade, can have poor outcomes. These high rates of morbidity and mortality are mainly related to the extent of bleeding and other complications, including cerebral vasospasm. Clinical treatment has been largely ineffective at improving the outcome. A selective calcium channel blocker (nimodipine) remains the only evidence-based option in the management of subarachnoid hemorrhage, although with limited benefits. Conversely, delayed ischemic deficits represent the main source of neurological disability after cerebral aneurysm rupture, and prevention of cerebral arterial vasospasm constitutes a key target for new pharmacological treatments. Other options include statins and magnesium sulfate. However, recent trials have been unable to demonstrate any clinical benefits.

There is experimental and clinical evidence that sulfonylureas may be beneficial for patients with acute ischemic stroke. The pleiotropic neuroprotective effects of glibenclamide have been well substantiated over the past decade in clinically relevant models of human disease. Glibenclamide protects the microvascular endothelium to reduce the formation of edema and secondary hemorrhage, inhibits neuronal cell death, and exerts potent anti-inflammatory effects and promotes neurogenesis. Retrospective studies of patients with diabetes, as well as a recent Phase IIa pilot study in nondiabetic individuals, suggest a highly promising translational potential for therapeutic intervention with glibenclamide in ischemic stroke.

### Background information

Glibenclamide is a member of the sulfonylurea class of drugs and has been in clinical use as an oral hypoglycemic agent since the 1960s. Sulfonylurea drugs all work via a similar mechanism which is inhibition of the sulfonylurea receptor 1 (Sur1). Glibenclamide has received renewed attention due to its pleiotropic protective effects in acute central nervous system (CNS) injury [1–17]. In the CNS, glibenclamide exerts its effects primarily via inhibition of the recently characterized Sur1-Trpm4 channel (formerly, the Sur1-regulated nonselective cation (NC_Ca-ATP_) channel). Inhibition of Sur1 with glibenclamide has found to be an effective treatment in experimental models of various CNS pathologies, including ischemic and hemorrhagic stroke and subarachnoid hemorrhage [17–19].

### Experimental data

Experimentally, glibenclamide significantly reduced the mortality rate to 5% in models of ischemic stroke, whereas the vehicle-treated group had 67% mortality at 24 h. Compared to the decompressive craniectomy (DC) group, glibenclamide-treated rats exhibited less brain swelling at 24 h and improved neurological outcome that persisted for the 2 weeks of observation. Both DC and glibenclamide eliminated mortality, but neurological function during the next 2 weeks was significantly better with glibenclamide compared with DC [17].

### Clinical studies in ischemic stroke

Retrospective studies of patients with diabetes mellitus type II suffering from ischemic stroke suggest that being on a sulfonylurea drug and staying on it during hospitalization for stroke improves outcome and reduces the incidence of symptomatic hemorrhagic transformation and mortality [16, 17]. Recently, Phase II clinical trials have begun to evaluate an intravenous formulation of glibenclamide in patients with traumatic brain injury [20] and stroke [21–23]. The neuroprotective properties observed in the laboratory and in retrospective human studies have led to the initiation of prospective clinical trials in ischemic stroke and traumatic brain injury. These prospective trials are evaluating an injectable formulation of glibenclamide (RP-1127; Remedy Pharmaceuticals, Inc., New York, NY, USA). Recently, a two-center, prospective, open label, Phase IIa pilot study of RP-1127 was completed (ClinicalTrials.gov identifier NCT01268683) [23]. This clinical trial tested the effect of RP-1127 in 10 patients with a severe anterior circulation ischemic stroke at high risk for malignant cerebral edema. The incidence of malignant edema was 20%, compared with 88% in a prospective observational study of patients. Moreover, 8/10 patients did not require osmotherapy, intubation, or DC.

### Glibenclamide and experimental subarachnoid hemorrhage

The effect of glibenclamide was examined in a model of subarachnoid hemorrhage [19]. Twenty-four hours after injury, subarachnoid hemorrhage caused a large increase in blood–brain barrier permeability and disrupted the normal junctional localization of the tight-junction protein zona occludens (ZO-1). Glibenclamide significantly reduced ZO-1 abnormalities, resulting in less edema formation. In addition, subarachnoid hemorrhage led to large increases in several markers of inflammation, including tumor necrosis factor α and nuclear factor-κB, and markers of cell injury or cell death, including immunoglobulin G endocytosis and cleavage of caspase-3. Glibenclamide significantly reduced these effects as well. Glibenclamide considerably reduced transsynaptic apoptosis of hippocampal neurons, reduced venous congestion and significantly ameliorated impairments in spatial learning [16, 18].

### Clinical use of glibenclamide in subarachnoid hemorrhage

Glibenclamide has not been tested in the clinical treatment of subarachnoid hemorrhage thus far. Inflammation and delayed ischemic deficits account for a great deal of postaneurysmal rupture complications. The biological properties of glibenclamide may counterbalance inflammatory and ischemic consequences of subarachnoid hemorrhage, leading to better clinical outcomes. We believe that a clinical trial testing the effects of glibenclamide on the outcomes of patients with subarachnoid hemorrhage is currently fully supportable.

## Study goals and objectives

Thus far, there is no clinical study to evaluate the potential benefits of glibenclamide after aneurysmal subarachnoid hemorrhage. The time delay of 3 to 10 days until cerebral vasospasm develops after aneurysm rupture offers the unique opportunity to intervene before ischemia occurs. In this application, we outline a proposal to investigate the impact of the use of glibenclamide on the outcome of patients with aneurysmal subarachnoid hemorrhage. This information has major relevance, since subarachnoid hemorrhage is a prevalent condition and is a major cause of neurological disability and death. The lack of effective pharmacological measure to improve outcome in this scenario may be fulfilled by glibenclamide, with a significant medical and socioeconomic impact.

### Primary objective

The primary objective is to evaluate the role of glibenclamide on the clinical outcome of patients with aneurysmal subarachnoid hemorrhage. A prospective, randomized, double-blind trial was designed to evaluate the following hypothesis: glibenclamide improves clinical outcome after aneurysmal subarachnoid hemorrhage as measured by the modified Rankin scale (mRS).

### Secondary objectives

The secondary objectives are to assess the safety and effect of glibenclamide on the mortality rate, quality of life and cognitive performance in patients with aneurysmal subarachnoid hemorrhage. A prospective, randomized, double-blind trial was designed to evaluate the following hypothesis: glibenclamide decreases mortality and improves quality of life and cognitive performance in patients with aneurysmal subarachnoid hemorrhage.

## Study design

Glibenclamide in aneurysmatic subarachnoid hemorrhage (GASH) is a double-blind, prospective, randomized clinical trial. Recruitment will take place between 2018 and 2019 (Table 1).

## Methodology

### Study setting

This study is set in the Hospital das Clínicas, University of São Paulo, Brazil. Patients will be recruited from neurological/neurosurgical intensive and emergency care units.

### Eligibility criteria

Inclusion criteria will be radiological confirmatory evidence of an aneurysmal subarachnoid hemorrhage (by digital subtraction angiography, computed tomography angiography, or magnetic resonance angiography), age 18–70 years, and presentation less than 96 h from ictus. Exclusion criteria will be patients taking glibenclamide therapy at presentation, pregnancy, no reasonable prospect of survival (Hunt and Hess V), known renal or hepatic impairment, patient not fully independent before bleed, strong suspicion of drug or alcohol misuse, patient taking warfarin-type drugs, and suspected additional life-threatening disease.

### Intervention

Patients will be randomly assigned (1:1) to receive either glibenclamide 5 mg or placebo. Coded bottles containing either 21 similar tablets of glibenclamide 5 mg or placebo will be assigned a number. Patients will start treatment as soon as possible within 96 h of the ictus, with a daily dose until the 21st day after the bleed. Trial medication consists of one tablet a day, given orally or via a nasogastric tube. Aneurysm treatment, either by microsurgery or embolization, will be performed as soon as possible, according to the standard of care for the recruiting center. Nimodipine 60 mg, every 4 h, will be started on admission and continued till the 21st day after the ictus in all patients as recommended. No patient will be discharged during the time of treatment for a proper side effect monitorization.

**Fig. 1:**
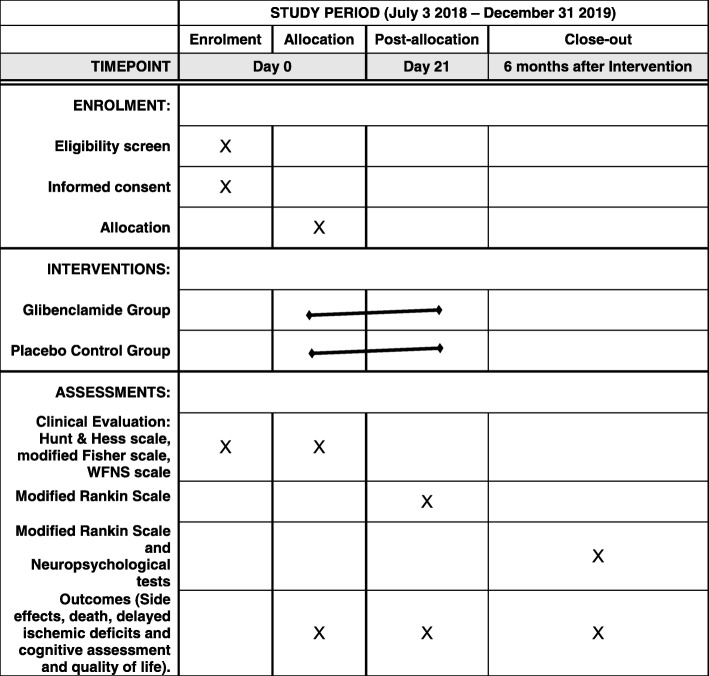
Schedule of enrolment, interventions, and assessments. WFNS World Federation of Neurological Societies

Patient demographics, medical history and relevant investigation results will be collected. The severity of the hemorrhage will be clinically assessed by the World Federation of Neurosurgical Societies grading scale and radiologically assessed using the modified Fischer scale. At 6 months of follow-up, patients will be analyzed with the mRS (a scale that measures degree of incapacity/dependence and mortality after neurological events) by a physician with no knowledge of treatment allocation. Neuropsychologists will also evaluate quality of life and cognitive performance using the Short-Form Health Survey Questionnaire (SF-36) and the Montreal Cognitive Assessment (MoCA) by their ten domains grade punctuation (0–100). We hypothesize that, compared with the control group, glibenclamide 5 mg will provide a better clinical outcome and additionally will decrease mortality and improve quality of life and cognitive performance after 6 months. Figure 1 shows the schedule of events.

**Table 1:** Trial duration

	2017	2018	2019	2020
Planning	+			
Recruitment		+	+	
Follow-up		+	+	+
Statistical analysis				+
Writing and submission				+

### Sample size

We estimated a sample size of 80 randomized patients to give 90% power at the 5% significance level (two-sided) to detect a treatment effect equivalent to an absolute increase of 7% in the proportion of patients with a favorable outcome (30-day mortality rate after subarachnoid hemorrhage approaches 40% and 25% of survivors present some degree of neurological morbidity [24]). This calculation was based on an ordinal analysis of the 6-month mRS (the primary outcome), assuming that the treatment effect follows a proportional odds model.

### Allocation

A computer-generated randomization code will be used to randomize medication bottles by blocks of ten (five glibenclamide, five placebo). Each bottle will be identified by a specific code number and subsequently selected for distribution in an ascending numerical order. All bottles are created, coded and stored at the study center’s pharmacy by one of the staff members who does not participate in the enrolment process.

One of the investigators will enroll recruited patients and assign them one of the random bottles. Drug administration will be provided by the nursing staff, who are also blinded, together with the rest of the patient’s other medications.

### Blinding

All participants, including patients, investigators, neuropsychologists and the center’s medical and nursing staff, are blinded and have no access to the meaning of the coding. The only exception to this is one of the investigators, who had prepared the coded bottles, who will not be blinded and will monitor possible adverse effects off the main drug, as discussed in more detail below. All patients have the right to withdraw at any stage of the study and learn their allocation.

### Safety considerations

An interim analysis will be performed when half of the sample size (40 patients) has completed the follow-up. An independent Data Monitoring Committee (DMC) will decide whether to stop or continue the trial after analyzing the evaluable data.

One of the investigators will have access to patient allocations to monitor possible side effects of glibenclamide, such as hypoglycemia. Capillary glycemia will be checked every 4 h during the 21-day treatment period and all measures to correct hypoglycemia will be taken. During the treatment time, no patients will be discharged.

The DMC will receive all data regarding the use of the drug and the possible side effects, as well the incidence of refractory hypoglycemia (serum glucose levels persistently < 70 mg/dL). If refractory hypoglycemia exceeds a frequency of 40% of treated patients, the trial will be interrupted. The same care will be taken for all other persistent events that are found.

### Follow-up

Patients will return after 6 months of follow-up to be evaluated by physicians and psychologists with no knowledge of the treatment allocation. Each patient’s mRS will be assessed by a physician, and psychologists will evaluate quality of life and cognitive performance using the SF-36 questionnaire and the MoCA test.

## Statistical analysis

The analysis of outcome measures will be on the intention-to-treat population, including those who died during the first 21 days. Results will be presented as an adjusted common odds ratio (OR) with a corresponding 95% confidence interval (CI), with values of the common OR of less than 1 indicating a treatment effect in favor of glibenclamide, with values of *p* < 0.05 considered statistically significant. Additionally, an analysis using logistic regression will be performed with the same covariates as specified above, adopting the conventional dichotomy of favorable mRS (0–2) versus unfavorable mRS (3–6). We will perform all analyses using the Mann Whitney *U* test, χ^2^ test or two-sample *t* tests as appropriate.

All statistical analysis will be performed with the SPSS Statistics for Windows software (IBM Corp., v. 24.0, Armonk, NY, USA). The analysis will also be blind for group allocations.

### Quality assurance

All data collected will be doubled checked by the study team and input into the REDCap system for storage and safe processing as a quality assurance measure.

### Expected outcomes

The primary outcomes will be the distribution of the mRS score assessed after 6 months of the end of the intervention, as favorable (mRS 0–2) and unfavorable (mRS 3–6). Secondary outcomes will be the cognitive assessment and quality of life as measured by the neuropsychological tests, as well as death, delayed ischemic deficits and the occurrence of serious adverse events attributable to medication. These secondary outcomes were preselected to provide supportive evidence related to the primary outcome.

The definition of delayed cerebral ischemia, as elaborated on by Vergouwen et al. [25], is taken as the occurrence of focal neurological impairment (such as hemiparesis, aphasia, apraxia, hemianopia, or neglect), or a decrease of at least 2 points on the Glasgow Coma Scale (either on the total score or on one of its individual components (eye, motor on either side, verbal)). This should last for at least 1 h, is not apparent immediately after aneurysm occlusion, and cannot be attributed to other causes by means of clinical assessment, computed tomography or magnetic resonance imaging of the brain, and appropriate laboratory studies. We will report any serious adverse events and those that were deemed to be related to the trial medication.

## Project duration and management

Patients will be evaluated by physicians at hospital admission, including intensive care physicians and neurosurgeons, to start complete management for subarachnoid hemorrhage. One of the researchers will apply the inclusion criteria and make the allocation with the randomly selected medication bottle. During the intervention, all adverse effects and neurological status will be recorded by the attending physicians. At the end of the follow-up period, physicians and the neuropsychologist will register the mRS and apply the neuropsychological tests. Once half of the sample is reached, one of the authors, previously selected by the safety committee, will have access to each patient’s allocation to determine the study’s continuity. At the end of the study, all data will be statistically analyzed for group comparisons.

## Discussion

This protocol evaluates the clinical effectiveness of glibenclamide in patients with acute brain aneurysmatic hemorrhage in a randomized, double-blind, placebo-controlled trial. Many other studies are already researching the effect of glibenclamide in brain edema and traumatic brain injury [17–23], but there is currently no study on aneurysmatic hemorrhage. More similar studies, like those of Jiang et al. [24] and Patel et al. [26], emphasized the positive effect of the drug in spatial memory and motor learning preservation in treated models of brain hemorrhage. The major goal of our trial is to establish the role of glibenclamide as an adjunct treatment in the scenario of posthemorrhagic delayed neurological onset. T﻿he limitations of this study may be related to potential confounding factors, including baseline clinical conditions and methods of aneurysm treatment (clipping or coiling). However, randomization may automatically match the groups and prevent confounding.

### Trial status

This is protocol version 2.1, 1 May 2019. This trial began recruitment on 3 July 2018 and currently has a total of 36 recruited patients. Most of these patients are still in the follow-up period and will soon be interviewed by a psychologist. The recruitment/allocation is anticipated to be complete by 1 August 2019, and the final follow-up should last until 31 December 2019. This submission includes the SPIRIT checklist as Additional file 1.

## Additional file

Additional file 1: SPIRIT 2013 checklist: recommended items to address in a clinical trial protocol and related documents. (DOCX 49 kb)

## Data Availability

All data generated and/or analyzed during this study will be included in the published article and its supplementary information files. All generated data, tables and statistical analysis will be available to the public via scientific publication.
